# Multi-Omics Analysis Reveals the Mechanism by Which *RpACBP3* Overexpression Contributes to the Response of *Robinia pseudoacacia* to Pb Stress

**DOI:** 10.3390/plants13213017

**Published:** 2024-10-28

**Authors:** Jian Zhou, Songyan Zhang, Pengxiang Die

**Affiliations:** 1 School of Horticulture and Landscape Architecture, Henan Institute of Science and Technology, Xinxiang 453003, China; zhangsy616@163.com (S.Z.); 17839577676@163.com (P.D.); 2 Henan Province Engineering Center of Horticulture Plant Resource Utilization and Germplasm Enhancement, Xinxiang 453003, China

**Keywords:** multi-omics, *RpACBP3* overexpression, *Robinia pseudoacacia*, Pb stress

## Abstract

Acyl-CoA-binding protein (*ACBP*) genes have been implicated in lead enrichment and translocation in plants; however, the mechanisms by which these genes contribute to the response to heavy metal stress in various taxa have not been determined. In this study, the molecular mechanisms underlying the response of *Robinia pseudoacacia*, an economically important deciduous tree, to Pb stress were examined using transcriptomic and metabolomic analyses. *RpACBP3* overexpression increased Pb enrichment, translocation, and tolerance. After Pb stress for 3 days, 1125 differentially expressed genes (DEGs) and 485 differentially accumulated metabolites (DAMs) were identified between wild-type and *RpACBP3*-overexpressing *R. pseudoacacia* strains; after Pb stress for 45 days, 1746 DEGs and 341 DAMs were identified. Joint omics analyses showed that the DEGs and DAMs were co-enriched in α-linoleic acid metabolism and flavonoid biosynthesis pathways. In particular, DEGs and DAMs involved in α-linoleic acid metabolism and flavonoid biosynthesis were up- and down-regulated, respectively. Moreover, *RpACBP3* overexpression enhanced the ability to scavenge reactive oxygen species and repair cell membranes under stress by regulating *LOX* gene expression and increasing the phosphatidylcholine content, thereby improving the tolerance to Pb stress. These findings lay a theoretical foundation for the future application of *RpACBP3 genes* in plant germplasm resource creation and phytoremediation of Pb contaminated soil in which *R. pseudoacacia* grow.

## 1. Introduction

Socioeconomic development and anthropogenic activities have resulted in increasingly severe heavy metal pollution, which can undermine the system stability of the soil and deteriorate the ecological function of the soil. As one of the most toxic heavy metals, second only to arsenic [1], Pb is mainly sourced from smelting and industrial solid waste emissions [2]. Pb does not degrade easily in soils, which can accumulate in plants through roots and ultimately enter human bodies through the food chain, posing severe hazards to human health, such as damaging the nervous system, blood system, and kidneys [3]. The remediation of lead contaminated soil is not only necessary but also urgent. To address Pb pollution in soils, phytoremediation is widely applied and has various advantages over other approaches, due to its low cost, environmentally friendliness, and large-scale applicability [4,5]. Woody plants have become a new direction for phytoremediation as they possess various advantages, such as large biomass, larger root systems than those of herbal plants, and a wide range of restoration applications [6].

*Robinia pseudoacacia*, a deciduous tree in the family Fabaceae, possesses an attractive shape, high aesthetic value, and strong resistance to drought, barrenness, salinity, alkalinity, and Pb pollution, making it an ideal tree species for environmental remediation [7,8]. Under Pb stress, *R. pseudoacacia* maintains stable physiological properties [9,10]. In preliminary analyses, we cloned an important Pb stress response factor, *Acyl-CoA-binding protein 3* gene of *R. pseudoacacia* (*RpACBP3*), with a total length of 1626 bp (Table S1) and a coding sequence of 1032 bp (Table S2), encoding 343 amino acids (Table S3) [11]. Acyl-CoA-binding proteins (ACBPs) are a family of proteins with highly conserved acyl-CoA-binding domain that can bind and transport various acyl CoA esters [12]. The plant ACBP proteins are divided into four categories: small ACBP (Class I); ankyrin repeat *ACBP* (Class II); large ACBP (Class III); and kelch ACBPs (Class IV) [13]; the RpACBP3 protein belongs to Class III. Plant *ACBPs* are involved in biosynthesis, growth and development, and membrane lipid metabolism as well as biotic and abiotic stress response and constitute an important class of stress resistance genes [14]. For example, after silencing the *TaACBP4A* gene, wheat showed increased sensitivity to powdery mildew [15], and overexpression of *OsACBP4* enhanced salt tolerance in rice [16]. *AtACBP1* overexpression strengthens Pb enrichment and translocation in *Arabidopsis thaliana*, and *atacbp1* mutants are more sensitive to Pb stress [17]. *AtACBP2* overexpression strengthens cadmium (Cd) and H_2_O_2_ tolerance in *A. thaliana* and promotes Cd translocation in the roots [18]. Additionally, *AtACBP1* and *AtACBP4* in *Brassica juncea* increase resistance to Pb stress, promote Pb enrichment in root tips, and increase cytosol contents of fibrovascular tissue [19]. These findings indicate that plant *ACBPs* have great potential in the phytoremediation of heavy metal-contaminated soil. However, few studies have evaluated the mechanisms by which *ACBP* mediates plant responses to heavy metal stress. The pathway and mechanism by which *RpACBP3* regulates Pb tolerance in *R. pseudoacacia* are unclear.

In recent years, multi-omics analyses have been applied extensively in studies on plant genetics and metabolic processes. Based on joint transcriptomic and metabolomic analyses, Xiong et al. [20] argued that *CitCHS* is a key factor for flavonoid synthesis in sweet orange ‘Newhall’ peels under Mg-deficient conditions. Joint transcriptomic and metabolomic analyses have revealed that exogenous fulvic acids enhance tolerance to drought stress in tea trees by regulating ascorbic acid metabolism, glutathione metabolism, and flavonoid biosynthesis [21]. Black mesh coverings can strengthen cold tolerance in pineapples by regulating flavonoid biosynthesis, phenylalanine metabolism, valine, leucine and isoleucine degradation, and diphenylethylene, diarylheptanoid, and gingerol biosynthesis [22]. In summary, multi-omics analyses have become a significant method to explore the mechanisms of plant response to adversity.

Using the *Agrobacterium*-mediated method, we homologously transformed *RpACBP3* into *R. pseudoacacia* and obtained *RpACBP3*-positive strains [11]. In this study, we aimed to conduct transcriptomic and metabolomic analyses of wild-type (WT) and *RpACBP3*-overexpressing *R. pseudoacacia* under Pb stress to explore differentially expressed genes (DEGs), differentially accumulated metabolites (DAMs), and important pathways involved in the response to Pb stress as well as to elucidate the molecular mechanism of *RpACBP3*-overexpression regulating the response of *R. pseudoacacia* to Pb stress.

## 2. Results

### 2.1. Relative Expression of RpACBP3 and Physiological Characteristics of Transgenic R. pseudoacacia Under Pb Stress

**Figure 1:**
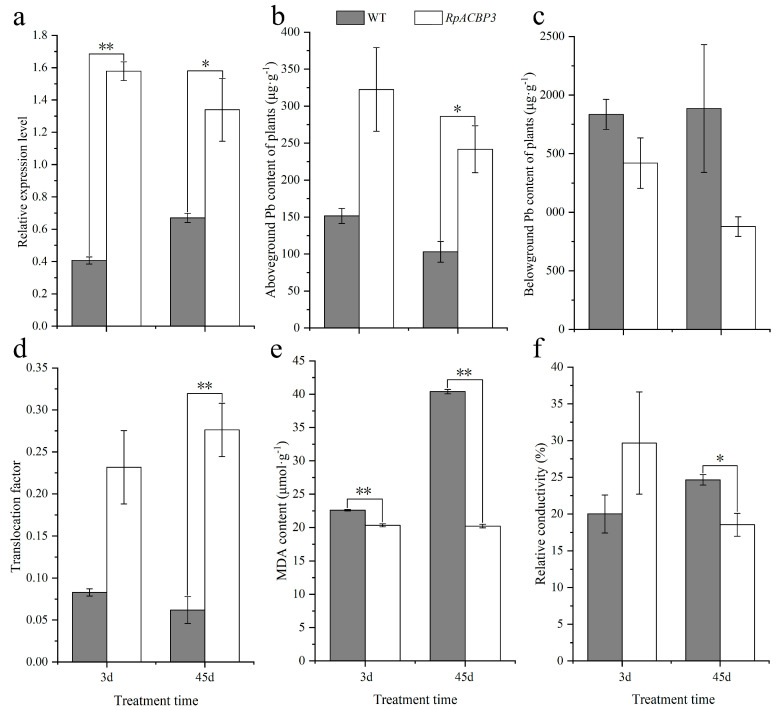
Relative expression of *RpACBP3* gene and physiological characteristics in transgenic and WT *Robinia pseudoacacia*. (**a**) Relative expression level of *RpACBP3* gene; (**b**) Pb content in aboveground plant; (**c**) Pb content in belowground plant; (**d**) Pb translocation factor in plant; (**e**) malondialdehyde (MDA) content; (**f**) relative conductivity (REC) of cytomembrane. WT: wild-type plants; *RpACBP3*: transgenic *RpACBP3*-positive lines OE5. The error bar in the figure represents the standard deviation (*n* = 3), while **, * indicate significant differences between transgenic and wild-type lines at the levels of *p* < 0.01 and *p* < 0.05, respectively, by Student *T*-test.

Under 3 and 45 days of Pb stress, *RpACBP3* gene expression levels in transgenic *R. pseudoacacia* were highly significantly (*p* < 0.01) and significantly (*p* < 0.05) higher than those in WT strains (Figure 1a), indicating successful overexpression in the OE5 strain. Both the aboveground Pb content and translocation factor (TF) of transgenic *R. pseudoacacia* under Pb stress were significantly higher than those in WT strains (*p* < 0.05) (Figure 1b,d) under 45 days of stress. The belowground Pb content of transgenic *R. pseudoacacia* under Pb stress was lower than that in WT plants; however, the difference was not significant (*p* > 0.05) (Figure 1c). Additionally, the malondialdehyde (MDA) content of transgenic *R. pseudoacacia* under 3 and 45 days of stress were significantly lower (*p* < 0.01) than those in WT strains (Figure 1e). The relative membrane conductivity of transgenic *R. pseudoacacia* under 3 days of stress did not differ significantly (*p* > 0.05) from that in WT plants, whereas the relative conductivity (REC) of cytomembrane in transgenic *R. pseudoacacia* under 45 days of stress was significantly lower (*p* < 0.05) than that in the WT strain (Figure 1f). These results indicated that *RpACBP3* overexpression promoted Pb accumulation and translocation in *R. pseudoacacia*, reduced membrane lipid peroxidation, and maintained the integrity of the cytomembrane under Pb stress.

### 2.2. Transcriptome Analysis

#### 2.2.1. Transcriptome Data Quality and DEG Analysis

Third-generation sequencing of the *R. pseudoacacia* transcriptome generated 303,503 full-length transcribed sequences, and 54,435 unigenes were generated after the elimination of redundancy, with lengths ranging from 62 to 11,211 bp (Figure S1). Q_20_ values of all samples were greater than or equal to 95.74%, Q_30_ values were greater than or equal to 90.68%, and GC percentages were all approximately 44%, indicating that the sequencing data were of sufficient quality for further analyses (Table S4).

**Figure 2:**
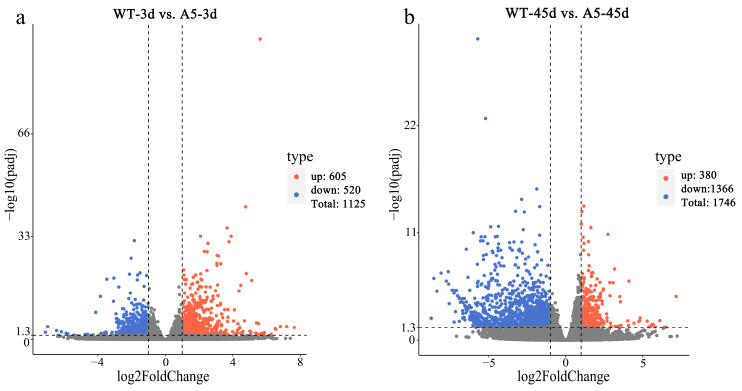
Differentially expressed genes (DEG) between transgenic and wild-type (WT) strains under Pb stress. (**a**) WT-3 d_vs_A5-3 d; (**b**) WT-45 d_vs_A5-45 d. WT-3 d_vs_A5-3 d represents the comparison group of WT and *RpACBP3* overexpression line of *Robinia pseudoacacia* under Pb stress for 3 days, while WT-45 d_vs_A5-45 d represents the comparison group of WT and *RpACBP3* overexpression line of *R. pseudoacacia* under Pb stress for 45 days.

In total, 1125 DEGs were identified between WT and *RpACBP3*-overexpressing *R. pseudoacacia* under 3 days of Pb stress, including 605 up-regulated and 520 down-regulated DEGs in *RpACBP3*-overexpressing *R. pseudoacacia* (Figure 2a). Additionally, 1746 DEGs (including 380 up-regulated and 1366 down-regulated DEGs) were identified between *RpACBP3*-overexpressing *R. pseudoacacia* under 45 days of Pb stress and the WT strain (Figure 2b).

#### 2.2.2. Kyoto Encyclopedia of Genes and Genomes (KEGG) Enrichment Analysis of DEGs

**Figure 3:**
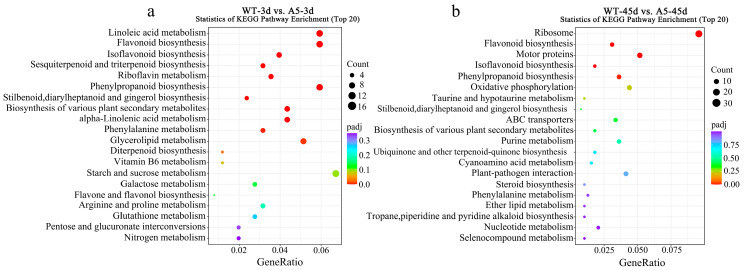
KEGG pathway enrichment results for differentially expressed genes. (**a**) WT-3 d_vs_A5-3 d; (**b**) WT-45 d_vs_A5-45 d. The size of the dots indicates the number of genes on the enrichment; the color indicates the false discovery rate (FDR) value, the lower the more significant; the red dots indicate the metabolic pathways with significant enrichment in both groups.

KEGG enrichment analysis of all DEGs was performed to identify the top 20 metabolic pathways with significant enrichment (*p* < 0.05). Under 3 days of Pb stress, DEGs were significantly enriched in linoleic acid metabolism, biosynthesis of flavonoids, isoflavones and propiophenones, and fat metabolism pathways (*p* < 0.05) (Figure 3a). Under 45 days of Pb stress, DEGs were significantly enriched in the biosynthesis of flavonoids, movement proteins, isoflavones, and propiophenones (*p* < 0.05) (Figure 3b).

**Figure 4:**
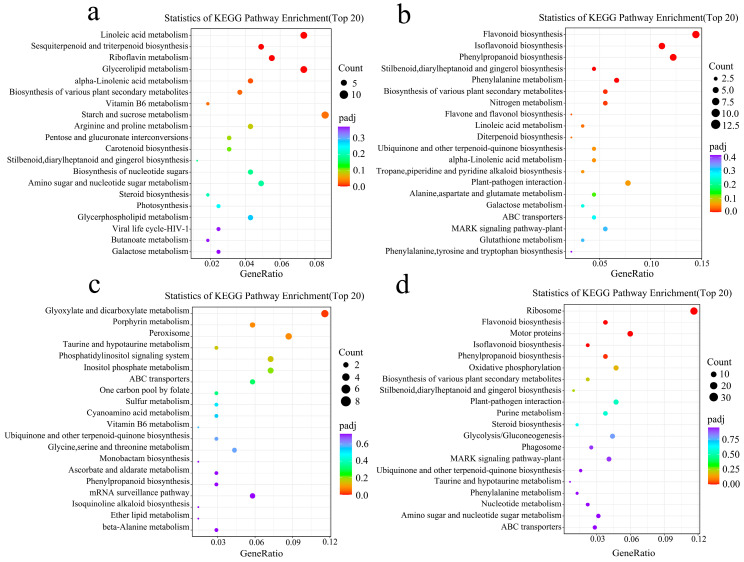
Metabolic pathways associated with up- and down-regulated genes. (**a**) Up-regulated metabolic pathways of WT-3 d_vs_A5-3 d; (**b**) down-regulated metabolic pathways of WT-3 d_vs_A5-3 d; (**c**) up-regulated metabolic pathways of WT-45 d_vs_A5-45 d; (**d**) down-regulated metabolic pathways of WT-45 d_vs_A5-45 d.

The up-regulated DEGs in *RpACBP3*-overexpressing *R. pseudoacacia* under 3 days of Pb stress were involved in metabolic pathways of starch and sucrose, linoleic acid, and fats (*p* < 0.05) (Figure 4a); significantly down-regulated DEGs were involved in the biosynthetic pathways of flavonoids, isoflavones, flavones, and flavonols *(p* < 0.05) (Figure 4b). The up-regulated DEGs in *RpACBP3*-overexpressing *R. pseudoacacia* under 45 days of Pb stress were related to the metabolic pathways of glyoxylate and dicarboxylate (*p* < 0.05) (Figure 4c), while down-regulated genes were involved in the biosynthetic pathways of propiophenones, flavonoids, and isoflavones *(p* < 0.05) (Figure 4d).

#### 2.2.3. Real-Time Quantitative Reverse Transcription PCR (RT-qPCR) Validation

**Figure 5:**
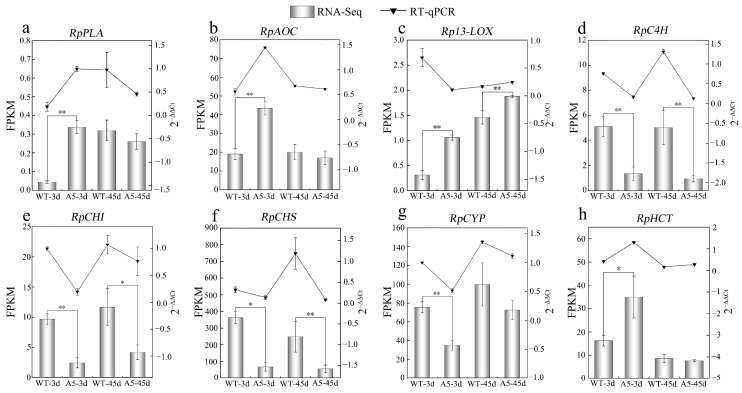
RT-qPCR validation and fragments per kilobase per million (FPKM) value of *RpPLA*, *RpAOC*, *Rp13S-LOX*, *RpC4H*, *RpCHI*, *RpCHS*, *RpCYP*, and *RpHCT* genes (**a**–**h**) in wild-type (WT) and transgenic *Robinia pseudoacacia* under Pb stress. The error bar in the figure represents the standard deviation (*n* = 3), while **, * indicate significant differences between transgenic and wild-type lines at the levels of *p* < 0.01 and *p* < 0.05, respectively, by Student *T*-test.

RT-qPCR was used to validate the results for genes identified in the lipid and flavonoid synthesis pathways. The variation tendencies of relative expression levels of *RpPLA*, *RpAOC*, *Rp13-LOX*, *RpC4H*, *RpCHI*, *RpCHS*, *RpCYP,* and *RpHCT* were nearly consistent with those of the fragments per kilobase per million (FPKM) values (Figure 5a–h). These results indicated that the transcriptome sequencing results were reliable.

### 2.3. Metabolome Analysis

#### 2.3.1. Metabolome Data Quality and DAM Analysis

During the metabolome analysis, correlations between samples were high within each group, with *R*^2^ values ranging from 0.814 to 1.000 (Figure S2a). In a principal component analysis (PCA), PC1 and PC2 accounted for 34.1% and 18.9% of the total variance, respectively, and effectively separated treatment groups (Figure S2b). A clustering heatmap showed that the homogeneity between samples was high within each group, whereas the heterogeneity between samples was high across different groups (Figure S2c), indicating the high stability of the detection process and high data quality.

**Figure 6:**
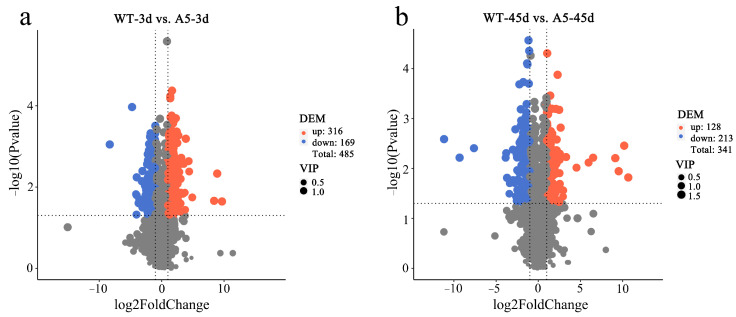
Differential metabolite volcano plots for different comparison groups. (**a**) WT-3 d_vs_A5-3 d; (**b**) WT-45 d_vs_A5-45 d. Horizontal coordinates indicate the fold change in metabolite differences across subgroups and vertical coordinates indicate the level of significance of the differences; each dot represents a metabolite, with significantly up-regulated metabolites represented by red dots and significantly down-regulated metabolites represented by blue dots; the size of the dot represents the VIP value, the larger the value the higher the contribution rate, the same below.

In total, 485 DAMs (*p* < 0.05) were identified between *RpACBP3*-overexpressing *R. pseudoacacia* under 3 days of Pb stress and WT plants, including 316 significantly up-regulated and 169 significantly down-regulated metabolites (Figure 6a). Additionally, 341 DAMs (including 128 significantly up-regulated and 213 significantly down-regulated metabolites) (*p* < 0.05) were identified between *RpACBP3*-overexpressing *R. pseudoacacia* under 45 days of Pb stress and WT plants (Figure 6b). These DAMs mainly covered amino acids, polypeptides, carbohydrates, fatty acids, lipids, organic acids and derivatives, phenylpropanoids, and polyketides.

#### 2.3.2. KEGG Enrichment Analysis of DAMs

**Figure 7:**
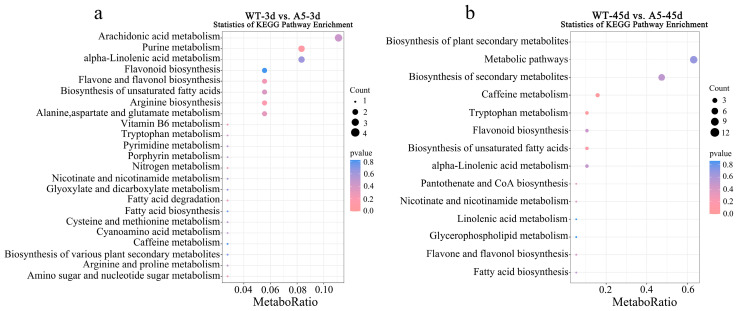
KEGG pathway enrichment of differential metabolites in different comparison groups, (**a**) WT-3 d_vs_A5-3 d; (**b**) WT-45 d_vs_A5-45 d. The size of the point represents the number of metabolites on the enrichment; the lower the *p* value represented by color, the more significant; the red font represents the up-regulated pathway, and the blue font represents the down-regulated pathway.

KEGG enrichment analysis was performed to evaluate all DAMs. *RpACBP3* overexpression altered the metabolism of arachidonic acids, purines, and α-linoleic acid and biosynthesis of flavonoids, flavones, flavonols, and unsaturated fatty acids in *R. pseudoacacia* under 3 days of Pb stress; however, the alterations were not significant (*p* > 0.05) (Figure 7a). *RpACBP3* overexpression significantly altered the metabolism of tryptophan and caffeine and biosynthesis of plant secondary metabolites in *R. pseudoacacia* under 45 days of Pb stress (*p* < 0.05) (Figure 7b). During the Pb stress period, metabolites in the biosynthetic pathways of flavonoids, flavones, flavonols, unsaturated fatty acids, and fatty acids were down-regulated in transgenic *R. pseudoacacia*, while metabolites in the metabolic pathways of α-linoleic acid were up-regulated under only 3 days of Pb stress. These findings were basically consistent with the KEGG enrichment analysis of DEGs.

### 2.4. Joint Analysis of Transcriptome and Metabolome 

#### 2.4.1. KEGG Co-Enrichment Analysis of DEGs and DAMs

**Figure 8:**
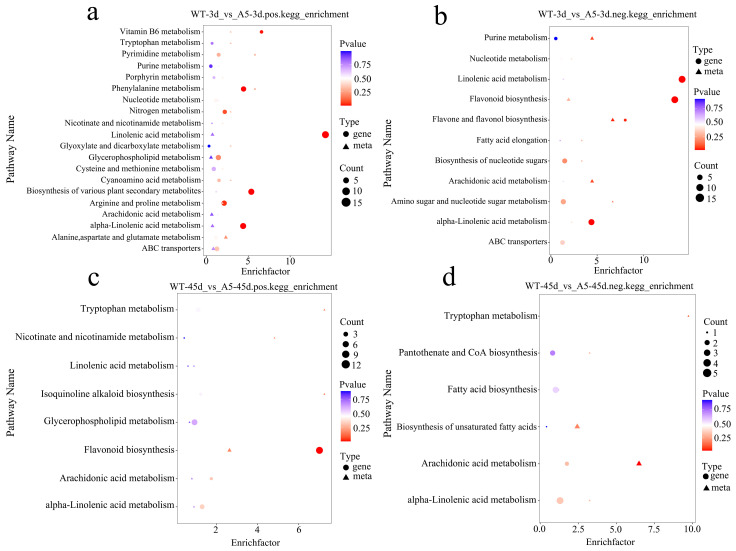
Co-enrichment analysis of differentially expressed genes (DEGs) and differentially accumulated metabolites (DAMs) using KEGG across different comparison groups. (**a**) Positive ion mode of WT-3 d_vs_A5-3 d; (**b**) negative ion mode of WT-3 d_vs_A5-3 d; (**c**) Positive ion mode of WT-45 d_vs_A5-45 d; (**d**) negative ion mode of WT-45 d_vs_A5-45 d. The gradient from red to purple represents the change in the significance of enrichment from high to low.

KEGG co-enrichment analysis was performed to evaluate the DEGs and DAMs in *R. pseudoacacia*. Under 3 days of Pb stress, DEGs and DAMs in transgenic *R. pseudoacacia* were most significantly co-enriched in the metabolic pathways of linoleic acid, α-linoleic acid, arginine, and proline and in the biosynthetic pathways of flavonoids and plant secondary metabolites (Figure 8a,b). Under 45 days of Pb stress, DEGs and DAMs in transgenic *R. pseudoacacia* were most significantly co-enriched in the biosynthetic pathways of flavonoids and in the metabolic pathways of glycerophospholipid, tryptophan, and linoleic acid (Figure 8c,d). DEGs and DAMs in *R. pseudoacacia* under 3 days of Pb stress shared numerous pathways, indicating that *RpACBP3* overexpression significantly affected metabolic processes in the initial stage of Pb stress. The metabolism of α-linoleic acid and biosynthesis of flavonoids are co-enriched pathways in analyses of DEGs and DAMs during stress and should be important pathways for transgenic *R. pseudoacacia* to cope with Pb stress.

#### 2.4.2. Co-Expression Network Analysis of Important Metabolic Pathways

**Figure 9:**
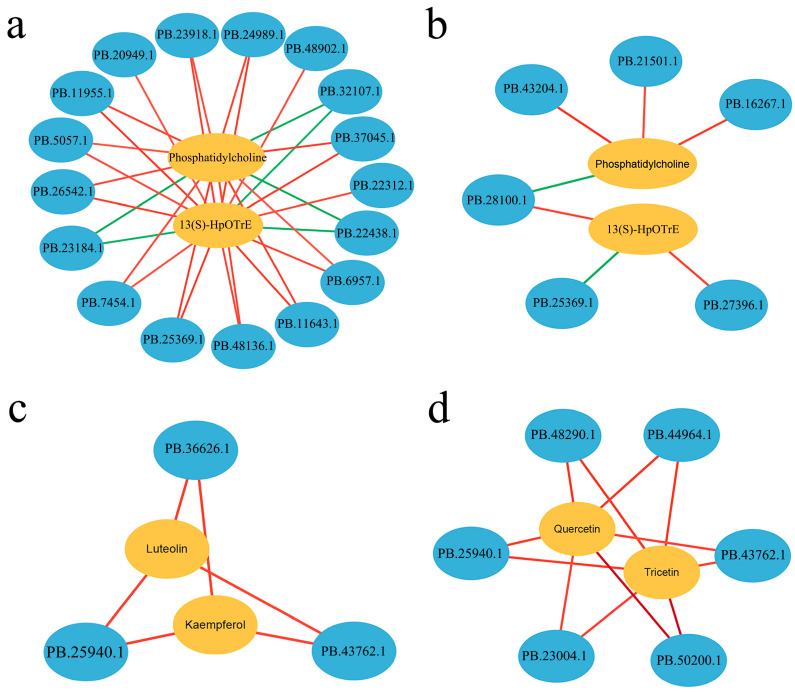
Co-expression network analysis of differentially expressed genes (DEGs) and differentially accumulated metabolites (DAMs) in important metabolic pathways in different comparison groups. The networks in α-linoleic acid metabolism in (**a**) WT-3 d_vs_A5-3 d, and (**b**) WT-45 d_vs_A5-45 d, and those in flavonoid biosynthesis in (**c**) WT-3 d_vs_A5-3 d, and (**d**) WT-45 d_vs_A5-45 d. Metabolites are represented in yellow, while genes are represented in blue. The connecting line between genes and metabolites represents correlation, with red indicating positive correlation and green indicating negative correlation.

Co-expression network analysis was performed to evaluate the DEGs and DAMs in the α-linoleic acid metabolism and flavonoid biosynthesis pathways. The α-linoleic acid metabolism pathways involved phosphatidylcholine (PC) and 13(S)-HpOTrE and were significantly correlated (predominantly positively correlated) with multiple genes (*p* < 0.05). Specifically, they were correlated with numerous genes under 3 days of Pb stress (Figure 9a) but with fewer genes under 45 days of Pb stress (Figure 9b). The flavonoid biosynthesis pathways, including kaempferol, luteolin, quercetin, and tricetin pathways, and were positively correlated with the associated genes. They were correlated with fewer genes under 3 days of Pb stress (Figure 9c) than under 45 days of Pb stress (Figure 9d), leading to a more complex co-expression network over time.

#### 2.4.3. Integrative Analysis of Important Metabolic Pathways

**Figure 10:**
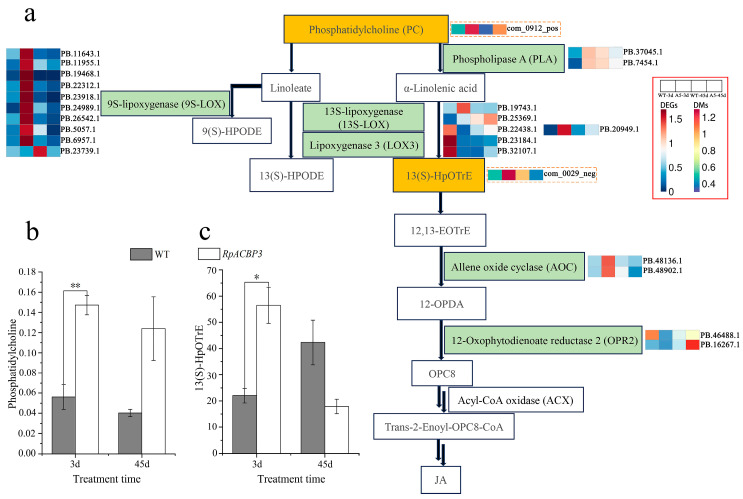
Integrated analysis of differentially expressed genes (DEGs) and differentially accumulated metabolites (DAMs) in α-linoleic acid metabolism pathway of transgenic *Robinia pseudoacacia* under Pb stress. (**a**) Metabolic pathways, (**b**) PC change during stress, and (**c**) changes in 13(S)-HpOTrE level during stress. The yellow box represents differential metabolites; the green box represents differential genes and enzymes encoded by differential genes; the red indicates up-regulation; and the blue indicates down-regulation. The error bar represents the standard deviation (*n* = 3), while **, * indicate significant differences between transgenic and wild-type lines at the levels of *p* < 0.01 and *p* < 0.05, respectively, by Student *T*-test in (**b**,**c**).

*RpACBP3* overexpression increased the PC and 13(S)-HpOTrE contents in the metabolic pathways of α-linoleic acid in transgenic *R. pseudoacacia* under Pb stress (Figure 10a) and up-regulated key genes significantly, such as phospholipase A (*PLA*), lipoxygenase (*LOX*), and allene oxide cyclase (*AOC*) (*p* < 0.05) under 3-day Pb stress, with no significant differences (*p* > 0.05) under 45 days of stress compared with levels in WT strains (Figure 5a–c and Figure 10b,c). PC was converted into 13(S)-HpOTrE under the action of enzymes, such as PLA, 13S-lipoxygenase (13S-LOX), and LOX3; subsequently, 13(S)-HpOTrE was catalyzed by AOC and 12-oxophytodienoate reductase 2 (OPR2) and metabolized by β-oxidation thrice, finally generating jasmonic acid (JA).

**Figure 11:**
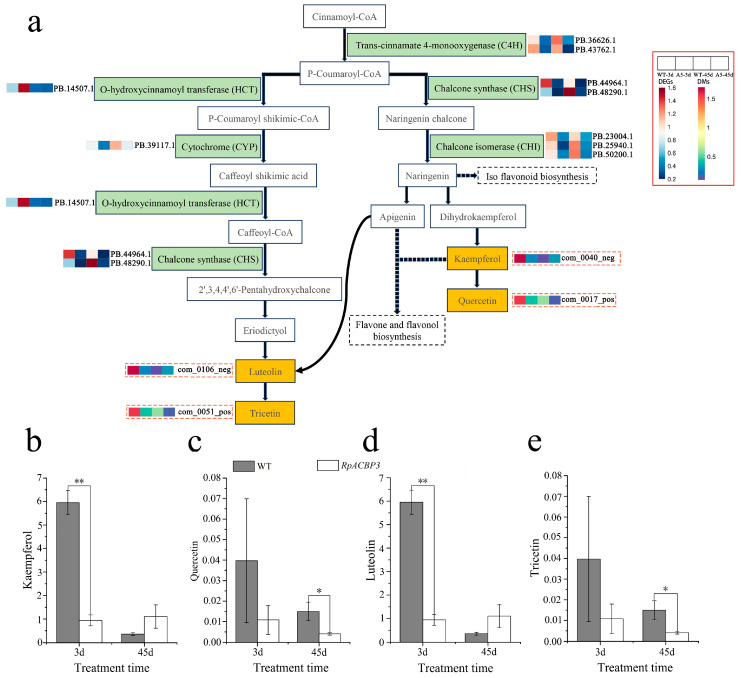
Integrated analysis of differentially expressed genes (DEGs) and differentially accumulated metabolites (DAMs) in flavonoid biosynthesis pathway of transgenic *Robinia pseudoacacia* under Pb stress. (**a**) Metabolic pathways, (**b**) Kaempferol change during stress, (**c**) Quercetin change during stress, (**d**) Luteolin change during stress, and (**e**) Tricetin change during stress. The yellow box represents differential metabolites; and the green box represents differential genes and enzymes encoded by differential genes; the red indicates up-regulation; and the blue indicates down-regulation in (**a**). The error line in the figure represents the standard deviation (*n* = 3), while **, * indicate significant differences between transgenic and wild-type lines at the levels of *p* < 0.01 and *p* < 0.05, respectively, by Student *T*-test in (**b**–**e**).

*RpACBP3* overexpression reduced the levels of DAMs in flavonoid biosynthetic pathways in transgenic *R. pseudoacacia* under Pb stress (Figure 11a). In particular, it significantly reduced the contents of kaempferol and luteolin under 3 days of Pb stress and the contents of quercetin and tricetin under 45 days of Pb stress (*p* < 0.05) (Figure 11b–e). Additionally, *RpACBP3* overexpression reduced the expression levels of most DEGs significantly (*p* < 0.05), such as chalcone synthase (*CHS*), trans-cinnamate 4-monooxygenase (*C4H*), cytochrome (*CYP*), and chalcone isomerase (*CHI*) (Figure 5d–g). The flavonoid biosynthesis pathways were relatively complex. Cinnamoyl coenzyme A was catalyzed by various enzymes, such as C4H, CHS, and CHI, ultimately generating flavonoid metabolites, such as kaempferol and quercetin. In another branch, cinnamoyl coenzyme A was catalyzed by enzymes, including *O*-hydroxycinnamoyl transferase (HCT), CYP, and CHS, ultimately generating luteolin and tricetin.

## 3. Discussion

### 3.1. Effect of RpACBP3 Overexpression on Physiological Properties of R. pseudoacacia Under Pb Stress

Heavy metals accumulate in plant roots; however, only a few are translocated to the aboveground parts of plants, thus alleviating toxicity, especially in plant leaves [23]. This translocation capacity is also an important indicator for measuring the phytoremediation of heavy metal-contaminated soil [24]. In this study, the aboveground Pb content and TF in transgenic *R. pseudoacacia* were both evidently higher than those in the WT strain, whereas the belowground Pb content in transgenic plants was lower than that in the WT strain, indicating that *RpACBP3* overexpression enhanced Pb enrichment and translocation in *R. pseudoacacia*. These results were similar to results for *AtACBP1*, *AtACBP2,* and *AtACBP4*, whose overexpression significantly enhances Pb and Cd tolerance and enrichment in plants [17,18,19]. Additionally, the MDA content and REC of cytomembranes denote the degree of lipid peroxidation and the cytomembrane integrity, making them important indicators of cell functions [25]. Under 45 days of Pb stress, the aboveground Pb content in transgenic *R. pseudoacacia* increased, while the MDA content and cytomembrane conductivity decreased evidently, indicating that *RpACBP3* overexpression enhanced the Pb tolerance of *R. pseudoacacia*, reduced oxidative toxicity of cytomembrane and contributed to the maintenance of cytomembrane integrity and cell function.

### 3.2. Effect of RpACBP3 Overexpression on α-Linoleic Acid Metabolites in R. pseudoacacia Under Pb Stress

Linolenic acid (LnA) and linoleic acid (LA) are unsaturated fatty acids, and both can initiate unsaturated fatty acid metabolism in plants [26]. PC is a substrate for generating LnA and LA and is a key cytomembrane component, able to promote cytomembrane repair and essential to maintaining the cell structure and cytomembrane stability [27]. For example, exogenous PC can enhance the tolerance of *Prunus persica* to drought and salt stress, protect cell integrity, and reduce cytomembrane damage [28,29]. Studies have shown that AtACBP6 protein can bind to PC to enhance low temperature tolerance in *A. thaliana* [30]. In this study, *RpACBP3* overexpression increased the PC content in transgenic *R. pseudoacacia* under Pb stress. It is possible that excess RpACBP3 protein bounds to PCs, and then participates in cytomembrane repair, the maintenance of cytomembrane integrity, and cytomembrane function, thus enhancing the Pb tolerance in transgenic *R. pseudoacacia*.

*RpACBP3* overexpression also increased the content of 13(S)-HpOTrE in *R. pseudoacacia.* 13(S)-HpOTrE can induce the biosynthesis of protease inhibitors (PIs) to respond to stress [31]. PIs can regulate the activity of endogenous proteins in animals and plants and possess strong oxidation resistance [32,33]. Increased levels of PI proteins can significantly improve the tolerance of transgenic *Cassia tora* and *Populus deltoides* to environmental stresses, such as salt alkali, drought, and heavy metals [34,35]. Therefore, the enhancement of Pb tolerance in transgenic *R. pseudoacacia* by *RpACBP3* overexpression may also be related to increased PI biosynthesis, responding to Pb stress by removing reactive oxygen species (ROS) through antioxidant pathways.

### 3.3. Effect of RpACBP3 Overexpression on Lipid Metabolism in R. pseudoacacia Under Pb Stress

Under adverse conditions, the ROS content in plants increases cumulatively, leading to reductions in photosynthetic activity, lipid peroxidation, protein oxidation, and enzyme activity [36]. *LOX* is a key enzyme in the metabolic pathways of stearic acid in plants and is closely related to stress resistance [37]. *Capsicum annuum LOX* (*CaLOX1*) overexpression in *A. thaliana* reduces lipid peroxidation damage, H_2_O_2_ accumulation, and enhances tolerance to drought and salinity stress [38]. Under drought stress, *TaLOX11-6A* overexpression in *Triticum aestivum* reduces the MDA content, improves superoxide dismutase (SOD) activity, and enhances the ROS scavenging ability [39]. *Malus domestica LOX* (*MdLOX3*) overexpression in *A. thaliana* can significantly increase the activity of peroxidases (e.g., POD and SOD) and catalases (e.g., CAT), thereby significantly reducing the MDA and H_2_O_2_ contents and enhancing the tolerance to Zn stress [40]. In this study, *RpACBP3* overexpression increased the expression levels of *9S-LOX* and *13S-LOX* significantly in *R. pseudoacacia* (*p* < 0.05), which should be attributed to enhancing ROS scavenging and tolerance to Pb stress through strengthening antioxidant enzyme pathway.

Plant *PLA* is involved in the response to stress [41]. The promoter sequences of *Sorghum bicolor PLA* include multiple adversity response elements [42]. Under drought stress, *MaPLA1-2D* in *Morus alba* is significantly up-regulated by abscisic acid induction [43]. However, the response to abiotic stress (e.g., drought and salinity stress) differs among PLA genes, as demonstrated in *Linum usitatissimum, Gossypium arboretum,* and *Gossypium hirsutum* [41,44]. There are few reports of the functions of plant *PLA* genes under stress [41], and their functions may be related to hormonal signaling. Additionally, *AOC* is a key gene for the biosynthesis of JA and is involved in plant response to stress [45]. The overexpression of AOC genes in *Glycine max* (including *GmAOC1* and *GmAOC5*), *Camptotheca acuminata* (*CaAOC),* and *Saccharum spontaneum* (*ScAOC1*) can significantly enhance the tolerance ability to abiotic stress of tobacco [45,46,47]. *Gossypium hirsutum* AOC (*GhAOC1)* overexpression can reduce cytomembrane damage and lipid peroxidation in *A. thaliana* under Cu stress and enhance Cu tolerance through the JA pathway [48]. In this study, *RpACBP3* overexpression evidently increased the expression levels of *PLA* and *AOC* in *R. pseudoacacia* at the initial stage of Pb stress but did not alter the JA content significantly, indicating that overexpression of *RpACBP3* did not activate JA synthesis, and showed very weak association with the JA signal pathway under Pb stress and that JA-induced stress resistance was weak to Pb damage.

### 3.4. Effect of RpACBP3 Overexpression on Flavonoid Biosynthesis in R. pseudoacacia Under Pb Stress

Flavonoids, a class of polyphenolic secondary metabolites [49], can alleviate adversity- or stress-induced oxidative damage by scavenging ROS in plants [50]. Under adverse conditions, plants typically exhibit ROS overproduction and increased flavonoid biosynthesis [51,52]. Under drought stress, the contents of the flavonoid metabolites (e.g., caffeic acid and apigenin) in *Allium sativum* seedlings are upregulated by factors of 2.76 and 1.73, respectively [53]. Under arsenic stress, the flavonoid content in *Rosmarinus officinalis* increases by 65% [54]. Under Pb stress, the MDA and flavonoid levels in *Medicago sativa* also increase significantly [55]. In this study, DAMs and DEGs in the flavonoid biosynthetic pathways in transgenic *R. pseudoacacia* evidently decreased under Pb stress. These findings indicated that the production and activity of flavonoids in ROS scavenging in transgenic *R. pseudoacacia* decreased, and the degree of lipid peroxidation and cytomembrane integrity were superior in transgenic *R. pseudoacacia* than in WT strains. This could be attributed to the dominant role of other pathways of *R. pseudoacacia* in scavenging ROS and reducing the oxidative toxicity of Pb, such as the *LOX* gene and the 13(S)-HpOTrE-induced PI proteins, resulting in a lack of activation of flavonoid biosynthetic pathways. The mechanism of the decrease in flavonoid synthesis of *R. pseudoacacia RpACBP3* overexpression strains under lead stress, and further exploration is needed in later experiments.

## 4. Materials and Methods

### 4.1. Experimental Materials

WT *R. pseudoacacia* strains and *RpACBP3*-positive *R. pseudoacacia* strains (OE5 seedlings) (Figure S3) were provided by the Horticultural Plant Biotechnology Laboratory of School of Horticulture and Landscape Architecture, Henan Institute of Science and Technology.

### 4.2. Experimental Methods

#### 4.2.1. Experimental Design

The 3-month-old WT and OE5 strains were transplanted into cultivation pots (internal diameter, 14.8 cm), with one seedling cultivated in each pot, and 1.2 kg of cultivated soil (garden soil: nutrient soil: perlite: vermiculite = 3:1:1:1, *V*/*V*) accurately weighed in each pot. After 3 weeks, WT and OE5 were subjected to Pb(NO_3_)_2_ stress, with a Pb^2+^ concentration of 1500 mg·Kg^−1^, while the WT plants were used as the control group. Depending on the weight and the designed Pb concentration of the cultivated soil, the Pb(NO_3_)_2_ solution was placed in a tray under each pot, and the cultivated soil absorbed the Pb solution from the bottom to ensure even distribution of Pb^2+^ ions in the soil; the WT and OE5 strains were stressed for 3 and 45 days (named WT-3 d, WT-45 d, A5-3 d, and A5-45 d). Each treatment was repeated three times. Samples were then taken for physiological and omics analyses. The omics test samples were wrapped in tinfoil, flash-frozen in liquid nitrogen, and kept at –80 °C for transcriptome sequencing and a non-targeted metabolome analysis.

#### 4.2.2. RT-qPCR Analysis

Fresh leaves were taken from the WT and OE5 strains and ground into a powder using liquid nitrogen. Then, 0.1 g of powder was taken for total RNA extraction using the SteadyPure Plant RNA Extraction Kit (Accurate Biotechnology (Hunan) Co., Ltd., Changsha, China). Reverse transcription was conducted to synthesize cDNA using the Evo *M-MLV* Reverse Transcription Kit II (Accurate Biotechnology (Hunan) Co., Ltd., Changsha, China). RT-qPCR was conducted using the SYBR Green Pro Taq HS Premixed qPCR Kit (Accurate Biotechnology (Hunan) Co. Ltd.). For detailed procedures, refer to the instruction manuals of these kits. *Rp*Actin was used as the reference gene, and primers were designed using Primer Premier 5.0 (refer to Table S5 for the specific primer sequences). Relative gene expression was calculated using the 2^−ΔΔCt^ method [56], with three repetitions for each group.

#### 4.2.3. Physiological Determination

The malondialdehyde (MDA) level and REC were determined following the methods of Quan [57], with three replicates per group. The plant Pb ion concentration was determined using the method described in a previous study [58]. In particular, the samples were digested using a MASTER 40 microwave digestion instrument (Sineo Microwave Chemistry Technology (Shanghai) Co. Ltd., Shanghai, China), with three replications per group. TF represents the transport capacity of heavy metal ions in plants and refers to the transport of the ions from the underground part of the plant to the aboveground part in this study. The TF of Pb ion was calculated using the following equation.

TF = (Pb content in the aboveground part of the plant) ÷ (Pb content in the underground part of the plant)

#### 4.2.4. Transcriptome Sequencing Analysis

RNA extraction and sequencing were implemented by Bioyi Biotechnology Co., Ltd. (Wuhan, China). A cDNA library was constructed using the SMRTbell Template Prep Kit 1.0. After passing the quality check, samples were subjected to third-generation sequencing on the PacBio Sequel platform (Pacific Biosciences, Inc., Menlo Park, CA, USA). The raw sequencing data were filtered by IsoSeq analysis software (V4.0) (https://isoseq.how/ accessed on 2 February 2024), and some low-quality reads containing adapters were removed, resulting in hifi reads. Subsequently, their reads of inserts were analyzed, including the presence and positional relationships of 5′ primer, 3′ primer, and poly A, and classified to obtain the complete reads sequences containing 5′ primer, 3′ primer, and poly A with correct relative positions, known as Full-length sequences (FL reads). Subsequently, the terminal poly A sequences and chimeric sequences were removed to obtain the full-length transcripts. IsoSeq3 cluster was used for hierarchical alignment and iterative cluster merging of full-length transcripts, and redundant sequences were clustered together to obtain the consistent sequences. Finally, unigene sequences were generated by eliminating redundancies.

A cDNA library was constructed using an NEBNext Ultra II RNA Library Prep Kit. After passing the quality check, samples were subjected to next-generation sequencing on the DNBSEQ-T7 platform (MGI Tech CO., Ltd., Shenzhen, China). The raw data were filtered using FastTP (https://github.com/OpenGene/fastp accessed on 2 February 2024), removing all reads containing adapters, N ratios more than 10%, and low-quality base proportions greater than 50% (quality values less than 20). The clean reads were then subjected to quality control (QC) using FastQC (http://www.bioinformatics.babraham.ac.uk/projects/fastqc accessed on 2 February 2024). To meet the quality standards, Q_20_ values were required to exceed 90% and Q_30_ values to exceed 80%.

Gene expression levels were measured and normalized using the FPKM value. Differential gene expression was analyzed using DESeq2. DEGs were defined as follows: |Fold Change| greater than 2 and false discovery rate (FDR) lower than 0.05. DEGs were evaluated through KEGG annotation and KEGG pathway enrichment analyses using clusterProfiler (https://github.com/GuangchuangYu/clusterProfiler accessed on 2 February 2024), and FDR < 0.05 was the criterion for significant enrichment.

#### 4.2.5. Metabolomic Analysis

After samples were ground using liquid nitrogen, 100 mg was added to an Eppendorf Tube and supplemented with 500 μL of methanol solution (concentration: 80%). The sample was vortexed and vibrated and was kept in an ice bath for 5 min. Subsequently, it was centrifuged at 15,000× *g* and 4 °C for 20 min. A certain amount of supernatant was taken and diluted with mass spectrometry-grade water until the methanol concentration was 53%. The supernatant was centrifuged at 15,000× *g* and 4 °C for 20 min. The supernatant was collected and used for LC-MS. The sample was analyzed using a HypersilGold (C18) chromatographic column (Thermo Fisher Scientific, Waltham, MA, USA) at a flow rate of 0.2 mL/min and column temperature of 40 °C. In positive mode, 0.1% formic acid in mobile phase A and methanol in mobile phase B were used; in negative mode, 5 mM ammonium acetate in mobile phase A (pH value: 9.0) and methanol in mobile phase B were used. Mass spectrometry was conducted using a Q Exactive™ HF-X mass spectrometer (Thermo Fisher Scientific), with a spectral range of *m*/*z* 100 to 1500 *m*/*z*.

When processing raw data, the XCMS program was used for peak extraction, alignment, and retention time correction. The peaks with a missing rate >50% in each group of samples were filtered, the blank values were filled in with K-Nearest Neighbors algorithm, and the peak area was corrected using singular value decomposition method. The metabolites were identified by comparisons against the mzCloud (https://www.mzcloud.org accessed on 23 February 2024), mzVault, and Masslist databases. Based on the relative quantitative values of metabolites, the Pearson correlation coefficients between QC samples were calculated to reflect the stability of the detection process. Subsequently, PCA was performed on the total sample, and the stability of the method and data quality were reflected by differences within the sample group. The metabolite content data in the samples were standardized using unit variation scaling, and hierarchical cluster analysis was conducted on the metabolite accumulation patterns between different samples to reflect homogeneity within groups and heterogeneity between groups. Based on the above QC analyses, abnormalities can be found in a timely manner and problems can be solved early to ensure the quality of the experimental data.

The identified metabolites were annotated using the KEGG (https://www.genome.jp/kegg/pathway.html accessed on 23 February 2024), HMDB (https://hmdb.ca/metabolites accessed on 23 February 2024), and LIPID Maps (http://www.lipidmaps.org accessed on 23 February 2024) databases. Then, the metabolome data were processed using metaX, and the DAMs were identified according to the following criteria: VIP value (the first principal component in the OPLS-DA model) > 1, Fold Change >2.0 or <0.5, and *p* < 0.05. All DAMs were subjected to KEGG enrichment analysis, and *p* < 0.05 was used as the threshold for screening significant enrichment.

#### 4.2.6. Joint Transcriptome and Metabolome Analysis

During KEGG annotation, DEGs and DAMs assigned to the same pathway were selected to conduct a correlation analysis, and pathways with *p* < 0.05 were selected for a KEGG co-enrichment analysis. Subsequently, the correlations between DEGs and DAMs were evaluated using Pearson correlation coefficients. Correlated pairs were identified according to the following criteria: correlation coefficient| ≥0.80 and *p* < 0.05.

### 4.3. Statistical Analyses

Student *T*-test was conducted using SPSS 27.0, with a significance level α of 0.01 or 0.05. Histograms and line graphs were generated using Origin 2018. PCA analysis, OPLS-DA analysis, and correlation analysis were conducted with stats 4.0.3, mixOmics 6.14.1, and Hmisc 4.6.0 in R package, respectively. The relationship figures between DEGs and DAMs were visualized using Cytoscape 3.7.2. Cluster heatmap of metabolic pathways in joint analysis were generated using OmicStudio, a cloud-based platform of Lianchuan Biotech (LC-BioTechnologies (Hangzhou) Co., Ltd., Hangzhou, China).

## 5. Conclusions

**Figure 12:**
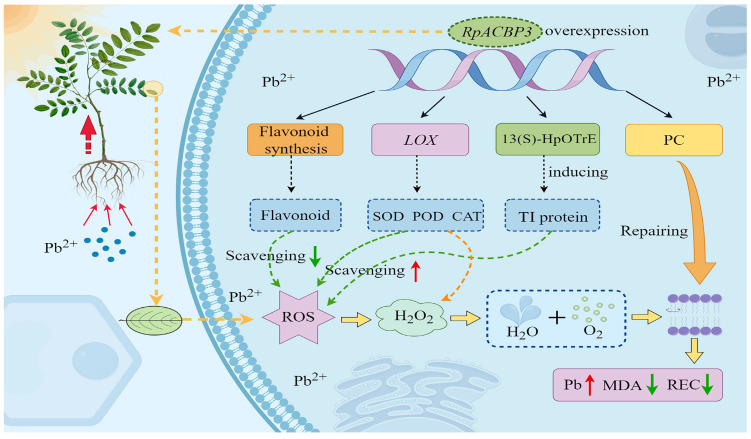
Proposed model of *RpACBP3*-overexpression regulating tolerance mechanisms of *Robinia pseudoacacia* to Pb stress. This model figure was drawn using FigDraw.

The findings of this study reveal that *RpACBP3* overexpression enhanced Pb enrichment, translocation, and tolerance in *R. pseudoacacia*. Joint transcriptome and metabolome analyses showed that DEGs and DAMs in *RpACBP3*-overexpressing *R. pseudoacacia* were co-enriched in pathways involved in α-LA metabolism and flavonoid biosynthesis. In particular, DEGs and DAMs involved in α-LA metabolism were upregulated, while those involved in flavonoid biosynthesis were down-regulated. It is proposed that *RpACBP3* overexpression may improve the ROS scavenging and cytomembrane repair abilities of *R. pseudoacacia* by regulating *LOX* and increasing 13(S)-HpOTrE and PC, thereby enhancing Pb tolerance (Figure 12). These findings show that the *RpACBP3* gene has a certain potential for application of *R. pseudoacacia* in plant remediation of Pb contaminated soil, which provides scientific basis for its later application into plant germplasm resource creation.

## Data Availability

The main results and Supplementary Data have already been presented in the manuscript, and the original data can be found below: https://www.ncbi.nlm.nih.gov/ accessed on 25 April 2024, PRJNA1104376.

## Supplementary Materials

The following supporting information can be downloaded at https://www.mdpi.com/article/10.3390/plants13213017/s1; Figure S1: Read length distribution of transcriptome Unigene of *Robinia pseudoacacia*; Figure S2: Quality analysis of metabolome data of *Robinia pseudoacacia* under Pb stress: (a) correlation heat map, (b) principal component analysis, and (c) cluster analysis; Figure S3: PCR detection of *Robinia pseudoacacia* transgenic strain, M: DNA marker DL 2000, P: positive plasmid control, N: negative control, WT-3 d, A5-3 d: WT and *RpACBP3* overexpression strain of *Robinia pseudoacacia* under Pb stress for 3 days, WT-45 d, A5-45 d: WT and *RpACBP3* overexpression strain of *Robinia pseudoacacia* under Pb stress for 45 days; Table S1: The full-length transcript sequence of *RpACBP3* gene; Table S2: The encoding sequence of *RpACBP3* gene; Table S3: The amino acid sequence of RpACBP3 protein; Table S4: Transcriptome sequencing data quality summary of *Robinia pseudoacacia*; Table S5: The primers used in this experiment.
