# Supplementary material for: Multi-Omics Analysis Reveals the Mechanism by Which RpACBP3 Overexpression Contributes to the Response of Robinia pseudoacacia to Pb Stress
Source: Plants (Basel). 2024 Oct 28;13(21):3017. doi: 10.3390/plants13213017 (PMC11548633; doi:10.3390/plants13213017)
Supplement: Supplementary file 1 [file plants-13-03017-s001.zip › Table S1.pdf]

Supplementary Table 1. The full-length transcript sequence of *RpACBP3* gene

Base sequence
ATGGGGATACAACCAAAGCATTAAGTGAAAAAAGCCGCCAGAAATAATGCTTTG
CTCTGTTTGCAGATTATCCTTTCCTCCTTTTTCCCTCATCTTCTCTTCCTTCCTTCTAG
TCATTTCCTTCCTAAATTCTCTCCTTTCTCTCTCTCTCTCTCTCTCTCCATGACAACC
ACAGTGTAATCCTCTGATCTTAATTCCCCTGTGCTTTAAACCTTTCCTCTCTCTCTCT
CTCTCACTTCACCCCATCACAACAAAAAGAAAGAAACAAAGAAGAAAGTGTTGT
CATGGAGCTTGTAACTGCAAGTGATCTTTTTGTCACTGCGTCGTTGGCGCTTATTTT
ATCATTCCTTGTTGCAAAGCTTGTCTCTTTGGCCATGACCGACACTCAAACCACGA
CAAACCATCATGTCTACGAGGAACCGGTTGGTCCGGTTCTTCATGGGGAACGGTT
CACCGTTCAGAGTAAGCATCAGTTCAATGACGAACCAGTTGGTCCGGTTCTTCAT
GGTGACCGGTACACGGTTCAAACAACACAAAGTGAAAGCAAGGTCGAATTTATT
AGTCCGGTTCAAGTTGCTACAATGAATGTAGAAGAAACCGGAGAGAATATAAAA
GAAGACGATACGGTTGAATTTGAATCGCCGGCGAAACCAGACATTGTCGTAGTCG
ATGAAATCAAAGAGAAAGAGAAAATCGCCGAGTCCAGTGACGATTCTACGGAAC
AGAGGAAAACGGAGTGTGTGGAAGAAATCATTGAAGAACCTTCCACTGAGGTTG
TAGTTTCCGTTGCAAAGGAAAAAGATGAGGAGAATGGTGATGATGATGATTGGGA
ATGGGAAGGGATTGAGAGGAGTGAGTTGGAGAAGGTGTTTATGGCGGCTACGGA
ATTTGTTGGTGTTGGTGGAAACGACGGTCGTTTTGGAAGCGATGTTCAGATGGAGT
TGTATGGGCTTCACAAGGTTGCTACCGAAGGACCTTGCCGTGAACCTCAACCAAT
GCCTCTCAAGCTCTCTGCACGTGCCAAGTGGAATGCTTGGCAAAAGTTGGGGAGC
ATGAGTCCAGAGGTTGCTATGGAGCAGTATATCAGCCTTCTTTCGGATAAAGTTCC
TGGATGGATGAAACATACTTCTTCTGCTGGAATGAGTGAACATGAACCTACGGGG
TCAGAAGTTTCTGAGCCTGCTGCTCCTGATTTGAGCACATCTTTGTCTCATCAACA
AATGATTGTAGCTGAAGGGGAACTTGAACAAAAGTCTGGTGCACAGAACCGTAG
CCTTCTTACCGAGTCAGATTTTGAGAACAATGTAAAGAAATGATGGCATCTCTCTG
CTTATATCCCGCTCCAAGGGACATTTTCCTGCATTGATTTCAGCAAAAAGGAGACT
GATTCCAAGAATAAGTTGGTTAGAAGTATGCCAGTTTCTTGGGCTGTTTGTTTTCTC
CTTTATGTTCTTCAATATCAAACTGTTAATATATATTTATTGTGCATAAATAAATGCG
TGTAAATATGATCTTATATGCTGCCGAAGGTTACATACTTCCTGGTTGTTGAAACTA
ATTTATTGATCTCATCATTCTCCTGTGTCCATTTAAGGTAGAAAAAAAAAAAAAAA
AAAAAAAAAAA
