# Supplementary material for: Multi-Omics Analysis Reveals the Mechanism by Which RpACBP3 Overexpression Contributes to the Response of Robinia pseudoacacia to Pb Stress
Source: Plants (Basel). 2024 Oct 28;13(21):3017. doi: 10.3390/plants13213017 (PMC11548633; doi:10.3390/plants13213017)
Supplement: Supplementary file 1 [file plants-13-03017-s001.zip › Table S2.pdf]

Supplementary Table 2. The encoding sequence of *RpACBP3* gene

Base sequence
ATGGAGCTTGTAACTGCAAGTGATCTTTTTGTCACTGCGTCGTTGGCGCTTATTTTA
TCATTCCTTGTTGCAAAGCTTGTCTCTTTGGCCATGACCGACACCCAAACCACGAC
AAACCATCATGTCTACGAGGAACCGGTTGGTCCGGTTCTTCATGGGGAACGGTTC
ACCGTTCAGAGTAAGCATCAGTTCAATGACGAACCAGTTGGTCCGGTTCTTCATG
GTGACCGGTACACGGTTCAAACAACACAAAGTGAAAGCAAGGTCGAATTTATTA
GTCCGGTTCAAGTTGCTACAATGAATGTAGAAGAAACCGGAGAGAATATAAAAG
AAGACGATACGGTTGAATTTGAATCGCCGGCGAAACCAGACATTGTCGTAGTCGA
TGAAATCAAAGAGAAAGAGAAAATCGCCGAGTCCAGTGACGATTCTACGGAACA
GAGGAAAACGGAGTGTGTGGAAGAAATCATTGAAGAACCTTCCACTGAGGTTGT
AGTTTCCGTTGCAAAGGAAAAAGATGAGGGGAATGGTGATGATGATGATTGGGA
ATGGGAAGGGATTGAGAGGAGTGAGTTGGAGAAGGTGTTTATGGCGGCTACGGA
ATTTGTTGGTGTTGGTGGAAACGACGGTCGTTTTGGAAGCGATGTTCAGATGGAGT
TGTATGGGCTTCACAAGGTTGCTACCGAAGGACCTTGCCGTGAACCTCAACCAAT
GCCTCTCAAGATCTCTGCACGTGCCAAGTGGAATGCTTGGCAAAAGTTGGGGAGC
ATGAGTCCAGAGGTTGCTATGGAGCAGTATATCAGCCTTCTTTCGGATAAAGTTCC
TGGATGGATGAAACATACTTCTTCTGCTGGAATGAGTGAACATGAACCTACGGGG
TCAGAAGTTTCTGAGCCTGCTGCTCCTGATTTGAGCACATCTTTGTCTCATCAACA
AATGATTGTAGCTGAAGGGGAACTTGAACAAAAGTCTGGTGCACAGAACCGTGG
CCTTCTTACCGAGTCAGATTTTGAGAACAATGTAAAGAAATGA
