# Supplementary material for: Multi-Omics Analysis Reveals the Mechanism by Which RpACBP3 Overexpression Contributes to the Response of Robinia pseudoacacia to Pb Stress
Source: Plants (Basel). 2024 Oct 28;13(21):3017. doi: 10.3390/plants13213017 (PMC11548633; doi:10.3390/plants13213017)
Supplement: Supplementary file 1 [file plants-13-03017-s001.zip › Table S3.pdf]

Supplementary Table 3. The amino acid sequence of RpACBP3 protein

Amino acid sequence
MELVTASDLFVTASLALILSFLVAKLVSLAMTDTQTTTNHHVYEEPVGPVLH<br>GERFTVQSKHQFNDEPVGPVLHGDRYTVQTTQSESKVEFISPVQVATMNVE<br>ETGENIKEDDTVEFESPAKPDIVVVDEIKEKEKIAESSDDSTEQRKTECVEEII<br>EEPSTEVVVSVAKEKDEGNGDDDDWEWEGIERSELEKVFMAATEFVGVGG<br>NDGRFGSDVQMELYGLHKVATEGPCREPQPMPLKISARAKWNAWQKLGS<br>MSPEVAMEQYISLLSDKVPGWMKHTSSAGMSEHEPTGSEVSEPAAPDLSTS<br>LSHQQMIVAEGELEQKSGAQNRGLLTESDFENNVKK
