# Supplementary material for: Multi-Omics Analysis Reveals the Mechanism by Which RpACBP3 Overexpression Contributes to the Response of Robinia pseudoacacia to Pb Stress
Source: Plants (Basel). 2024 Oct 28;13(21):3017. doi: 10.3390/plants13213017 (PMC11548633; doi:10.3390/plants13213017)
Supplement: Supplementary file 1 [file plants-13-03017-s001.zip › Table S4.pdf]

Supplementary Table 4. Transcriptome sequencing data quality summary of *Robinia pseudoacacia*

Sample	Total reads	Total bases	Clean reads	Clean bases	Q_{20} (%)	Q_{30} (%)	GC (%)
WT1-3d	44,115,936	6,617,390,400	44,096,600	6,425,713,150	96.65	92.61	44.32
WT2-3d	54,193,262	8,128,989,300	54,168,932	7,905,606,634	96.65	92.60	44.75
WT3-3d	66,254,312	9,938,146,800	66,215,130	9,648,009,216	95.74	90.68	44.64
WT1-45d	91,820,454	13,773,068,100	91,777,646	13,419,062,292	97.15	93.64	45.67
WT2-45d	43,056,252	6,458,437,800	43,035,266	6,283,346,884	96.85	92.93	44.60
WT3-45d	93,356,364	14,003,454,600	93,314,640	13,586,218,560	96.66	92.62	44.62
A5-1-3d	48,238,080	7,235,712,000	48,216,276	7,055,829,008	96.89	93.09	44.50
A5-2-3d	76,373,360	11,456,004,000	76,338,702	11,025,197,518	96.79	92.92	45.46
A5-3-3d	74,071,434	11,110,715,100	74,039,060	10,842,625,818	96.40	92.08	44.48
A5-1-45d	53,638,620	8,045,793,000	53,613,802	7,863,922,478	96.87	93.03	44.78
A5-2-45d	58,737,528	8,810,629,200	58,711,378	8,597,169,150	96.56	92.40	44.73
A5-3-45d	52,768,800	7,915,320,000	52,744,190	7,689,085,976	96.96	93.26	44.79
