# Supplementary material for: Multi-Omics Analysis Reveals the Mechanism by Which RpACBP3 Overexpression Contributes to the Response of Robinia pseudoacacia to Pb Stress
Source: Plants (Basel). 2024 Oct 28;13(21):3017. doi: 10.3390/plants13213017 (PMC11548633; doi:10.3390/plants13213017)
Supplement: Supplementary file 1 [file plants-13-03017-s001.zip › Table S5.pdf]

Supplementary Table 5. The primers used in this experiment

Primer name	Sequence（5′–3′）
RpActin	F：TTGCCTTGGATTATGAACA<br>R：GATGGCTGGAACAGAACTT
RpACBP3	F：ACCGGTACACGGTTCAAACA<br>R：GACAATGTCTGGTTTCGCCG
RpPLA	F：GCGGAGTGTACTTTCAAGGGT<br>R：CGAGAACACCTGCCAAGGAA
RpAOC	F：GCCCCATGCTGTTATTGCTG<br>R：AAACTTGAACCAAAGGTAGCA
Rp13-LOX	F：ACTGGTAGAGGGGTGCCTAA<br>R：TGTGGTAAACATGATCGGCG
RpC4H	F：CACTGAGAAAGGAGGGCAGTT<br>R：CCAGGGAGCACAGTGTAATCT
RpCHI	F：ATGGGGACTTATGGTGACGC<br>R：TGTGCCCTAAAGTTATCTGGAC
RpCHS	F：TGGTTTTGGACCTGGACTCAC<br>R：GGCTACCATTAGGCTACAAAGC
RpCYP	F：CGGTTCGGTTCAGGTGCTAT<br>R：CTTTAGCCAACTCCGCGTTC
RpHCT	F：CCAACATGGTACGCTGCAAG<br>R：AAGAATCTGCTCCACGGACG
