# Supplementary figures and images for: Multi-Omics Analysis Reveals the Mechanism by Which RpACBP3 Overexpression Contributes to the Response of Robinia pseudoacacia to Pb Stress
Source: Plants (Basel). 2024 Oct 28;13(21):3017. doi: 10.3390/plants13213017 (PMC11548633; doi:10.3390/plants13213017)

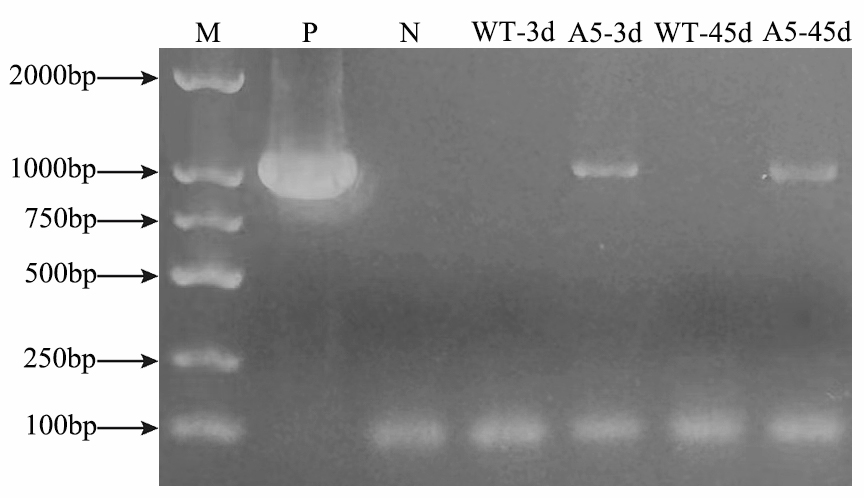

Supplement: Supplementary file 1 [file plants-13-03017-s001.zip › Figure S3.jpg]

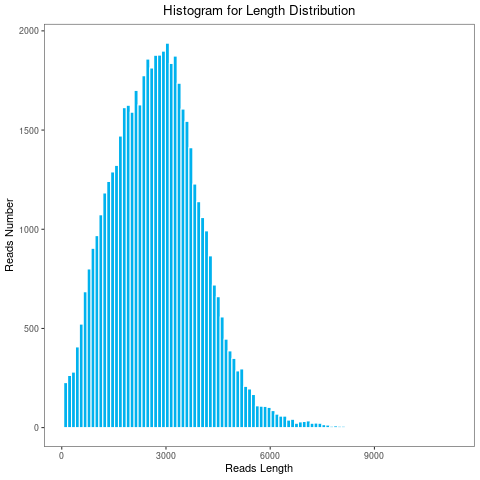

Supplement: Supplementary file 1 [file plants-13-03017-s001.zip › Figure S1.png]

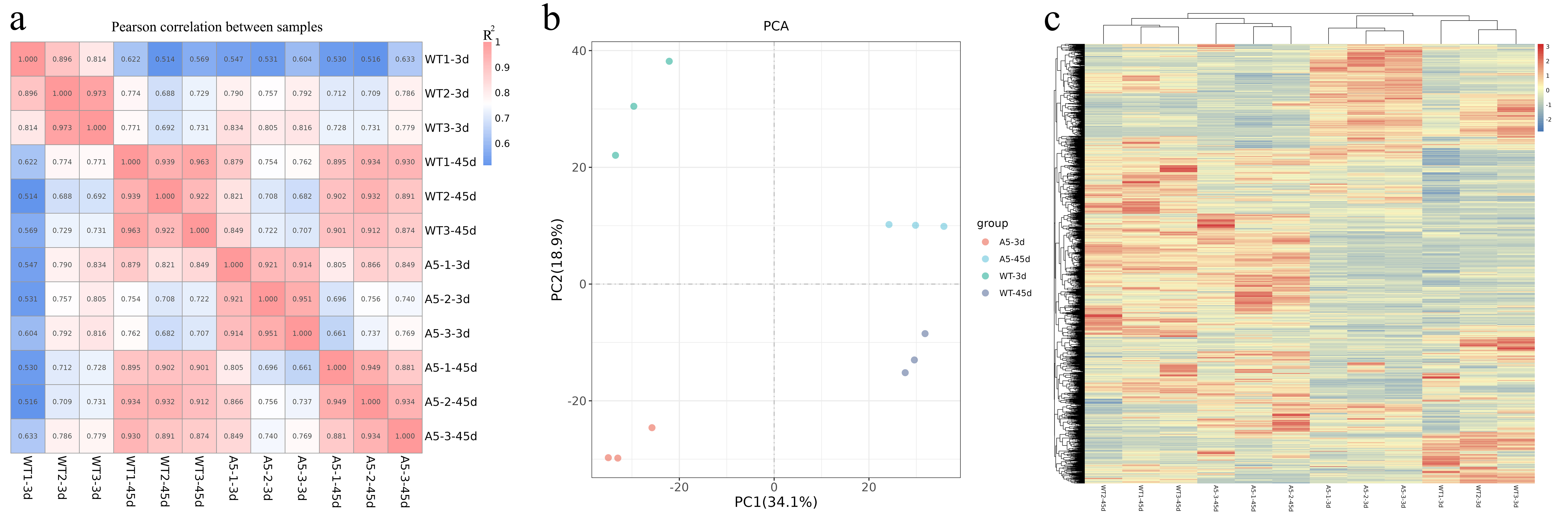

Supplement: Supplementary file 1 [file plants-13-03017-s001.zip › Figure S2.jpg]
